# Integration of Biobanks in National eHealth Ecosystems Facilitating Long-Term Longitudinal Clinical-Omics Studies and Citizens' Engagement in Research Through eHealthBioR

**DOI:** 10.3389/fdgth.2021.628646

**Published:** 2021-06-14

**Authors:** Athos Antoniades, Maria Papaioannou, Apostolos Malatras, Gregory Papagregoriou, Heimo Müller, Petr Holub, Constantinos Deltas, Christos N. Schizas

**Affiliations:** ^1^eHealth Lab, Department of Computer Science, University of Cyprus, Nicosia, Cyprus; ^2^biobank.cy Center of Excellence in Biobanking and Biomedical Research, University of Cyprus, Nicosia, Cyprus; ^3^Institute of Pathology, Medical University of Graz, Graz, Austria; ^4^Biobanking and Biomolecular Resources Research Infrastructure - European Research Infrastructure Consortium, Biobanks and Biomolecular Resources Research Infrastructure Consortium, Graz, Austria

**Keywords:** biobanks, ethical, national health, legal and social issues (ELSI), electronic health record

## Abstract

Biobanks have long existed to support research activities with BBMRI-ERIC formed as a European research infrastructure supporting the coordination for biobanking with 20 country members and one international organization. Although the benefits of biobanks to the research community are well-established, the direct benefit to citizens is limited to the generic benefit of promoting future research. Furthermore, the advent of General Data Protection Regulation (GDPR) legislation raised a series of challenges for scientific research especially related to biobanking associate activities and longitudinal research studies. Electronic health record (EHR) registries have long existed in healthcare providers. In some countries, even at the national level, these record the state of the health of citizens through time for the purposes of healthcare and data portability between different providers. The potential of EHRs in research is great and has been demonstrated in many projects that have transformed EHR data into retrospective medical history information on participating subjects directly from their physician's collected records; many key challenges, however, remain. In this paper, we present a citizen-centric framework called eHealthBioR, which would enable biobanks to link to EHR systems, thus enabling not just retrospective but also lifelong prospective longitudinal studies of participating citizens. It will also ensure strict adherence to legal and ethical requirements, enabling greater control that encourages participation. Citizens would benefit from the real and direct control of their data and samples, utilizing technology, to empower them to make informed decisions about providing consent and practicing their rights related to the use of their data, as well as by having access to knowledge and data generated from samples they provided to biobanks. This is expected to motivate patient engagement in future research and even leads to participatory design methodologies with citizen/patient-centric designed studies. The development of platforms based on the eHealthBioR framework would need to overcome significant challenges. However, it would shift the burden of addressing these to experts in the field while providing solutions enabling in the long term the lower monetary and time cost of longitudinal studies coupled with the option of lifelong monitoring through EHRs.

## Introduction

“It's more important to know what sort of person has a disease than to know what sort of disease a person has” is a phrase attributed to the ancient Greek physician Hippocrates. It is a phrase that was expressed almost 2,500 years ago and is one of the most powerful justifications for the necessity and the value of designing and developing complete, functional, and reliable electronic data and medical sample capture systems today that support medical research. It is clear, nowadays, that different types of persons require a different type of health management, either proactive or reactive (1). Being able to understand the type of person paves the way to select the most suitable and beneficial health strategy for the citizen's benefit. Thus, well-designed and implemented systems at the national level that include a well-integrated electronic health record (EHR) system to biobanks may be regarded as a tool for painting the full picture of a citizen's health status. This, in turn, will enable the discovery of specific disease subtypes that may be associated with specific treatment outcomes, empowering personalized healthcare and adapting preventive or therapeutic recommendations on the specialized health needs of every citizen.

By serving the citizen-centricity idealism, the central actor of the eHealth ecosystem is the citizen who is responsible for making the proper selections to improve his/her own health quality. Accordingly, it is crucial to allow the citizen to understand thoroughly the impact of choices at both the personal and societal levels, in terms of health and financial status. For example, citizens should become educated about the meaning of pharmacogenomics testing and how it can be used to minimize the ineffective medications and the ineffective doses as well as to control drugs' adverse effects (2–4). Human genome sequencing is an important biomedical finding, which generates an explosion of genetic data obtained for clinical purposes [e.g., spot medically actionable diseases or variants for which preventive measures are available (5, 6)]. The power of the analysis of this kind of genetic data can be further strengthened if it is incorporated into national integrated EHR systems (7, 8). The continuous dramatic drop in price of the genome sequencing escalates the need to find a way to accomplish this integration (9), and thus a mass adoption of sequencing-based technologies can be utilized for clinical care improvement (10). Notably, the genetic/genomics-based medicine is reported to reduce costs and improve outcomes mainly because of its preventive character, which allows the identification of potential or early-stage health problems (2).

Biobanks collect, catalog, and store biological samples, acting as a biorepository tool with great impact on medical research. Biobanks also act as databanks that maintain the data generated from the analyses of samples, providing researchers access to large numbers of digitized data across many subjects for often cross-purpose research studies. However, biobanks have provoked questions on privacy, research, and medical ethics. To address these challenges, biobanks adhere to governing principles and policies that ensure legal and ethical adherence of national-level (National Bioethics committees approvals) and European-level requirements [General Data Protection Regulation (GDPR)]. Digital means to support these activities have been created to minimize privacy risks and support research data sharing, which is part of the primary objective of biobanks.

EHRs are a systematized collection of patients' electronically stored health information in a digital format. EHR registries can exist in the institutional (hospitals and clinics) as well as national level. These are designed to store data accurately and to capture the state of the health of each person across time. The focus is not on the population but on the single individual patient; therefore, their direct application in research, although possible, is coupled with significant challenges. These are both technical (lack of homogeneity, missing data, etc.) and policy challenges (privacy legal/ethical) (11). They provide real-time digital citizen's health information records designed following the principles of citizen centricity. Consequently, all the actors of the healthcare community (healthcare providers, medical systems, medical organizations, etc.) should work as one around the citizen facilitating healthcare and improving treatment outcomes (4). To achieve that, the citizens have their own health data ownership, and they are given the power to control the access to their individual EHRs. Accordingly, healthcare providers will be given access to the most relative medical information by the citizen and the ability to create or maintain a citizen's EHR content data. In that way, the citizen will be able to dictate that his/her medical data follow him/her across the continuum of his/her care without any information discontinuities, loss of data, or communication problems between systems or people in a systematic fashion (8). Thus, EHR can be considered the backbone of a national eHealth ecosystem, which essentially joins the pieces of a citizen's health puzzle together.

An integrated national EHR system designed to cover the whole population and to be interoperable gives the right to the citizens to enjoy portable health, since EHR of a citizen will be remotely accessible by every healthcare provider across the country (12). To achieve that, a strong collaboration between IT and healthcare professionals is required (13) to define precisely and adequately EHR contents that will sufficiently embed workflows and automations reflecting the traditional applied clinical practice. As stated in (14), the process of rebuilding the EHR system from the ground up will be painful, but doing it properly “will make apparently insolvable problems solvable.” Furthermore, implementing such a system under the umbrella of the National eHealth Authority (NeHA) as explained in (12) will assure the standardization of the data elements and the architecture of the records (15) to be compliant with the EU directives and guidelines. Thus, accomplishing the primary target to develop a national eHealth ecosystem embracing all the healthcare actors under one standardized system will use a basic healthcare instrument in the integrated national EHR.

Many successful attempts have been made to capitalize on the wealth of data in EHRs for research purposes. These rely on focusing on a set number of specific attributes of interest to the research topic and on policies that need to be put in place to gain access to the data. The data attributes selected undergo quality control and transformations to meet homogeneity requirements before they are used in research. A common approach used to address privacy concerns is the pre-processing at the EHR registry side of the data to anonymize it, providing metadata that meet the research requirements while maintaining anonymity (k-anonymity approaches) (16, 17) and limiting the impact of privacy concerns. Others, instead, focus on federated analysis approaches where the analyses are run at each EHR registry with only the research outcomes made available to researchers. Even with high-dimensional genetic data, a data type inherently difficult to anonymize k-anonymity approaches can be applied to transform the data to be usable for research anonymized metadata (18).

## Methodology

The target of the proposed eHealthBioR framework is to facilitate the implementation of biomedical research by allowing the citizen to provide authorization to specific research studies that link the citizen's data sourced from a biobank and an EHR system. Citizens will be able to monitor the access to their data, and they will be able to withdraw their consent at any moment for any individual project. The eHealthBioR takes for granted that the biobank and the national integrated EHR system have collected and stored data in the corresponding databanks from the same individuals. The legal and ethical framework in which biobanks and the national integrated EHR system work internally is out of the scope of this paper. Rather, the target is to provide a framework schema that allows the citizens to have an active role in their participation in research studies combining data sourcing from a national biobank and EHR while providing the infrastructure to allow for the mechanisms to be compliant with GDPR, as well as with other national or international legal or ethical requirements.

### Current State-of-the-Art in Electronic Health Record–Biobank Integration

Biobanks can benefit the most from their integration with the national EHR by utilizing the wealth of detailed and longitudinal EHR data sources, which are enriched for clinically relevant phenotypes and outcomes to study genetics at a population scale (5, 19, 20). A nationwide EHR-coupled biobank also permits the reduction of the demographically distinct group bias, which underlies biomedical research studies allowing the easy and fast creation of large, inclusive patient/citizen cohorts that foster investigation of a biomedical hypothesis (3). Additionally, gaining access to nationalized pharmaceutical and cause of death registries can provide useful information for phenotype curation (19). Nevertheless, the integration of national EHR with biobanks will allow the latter to carry out long-term follow-up research studies, whereas the results will not be limited to diseases for which the participants were originally assessed (21) but also to the updated and current health status of the participants. Not only that, but in (21), cost and time efficiency of EHR-linked biobanks are reported in multiple ways using the BioVU, an EHR-based biobank paradigm. Although large in scale, BioVU is not linked to a nationwide EHR. Thus, we can safely conclude that the cost-saving infrastructure of an EHR-integrated biobank grows dynamically as the EHR population reflects the whole country's citizens. This effect is even larger when the EHR and the biobank are designed from scratch to serve each other and synergistically improving citizens' health quality and standards.

GDPR requires that data subjects should be able to determine in advance what the scope and the consequences of their data processing are and should not be taken by surprise at a later point about the ways in which their personal and health data have been used (22). Thus, the transparency of the processing can be characterized as forming the underlying basis for any exercise of the rights of the data subject. The eHealthBioR allows the citizen to grant access to both biobanking and clinical data to specific entities (e.g., researchers) in a transparent framework under which the citizen has the control to view, monitor, and (whenever desired) withdraw the access rights that he/she provides for specific research studies. The citizen authorizes researchers to process his/her data for very specific purpose(s) in the context of a research study. The authorization is granted for a very specific study, which solely allows the researcher to use it for this purpose. Any other project requires another separate authorization.

The efficiency of the aforementioned EHR-based biobank services will be further leveraged by the development of computational methods, which will accurately extract data from clinical databases and link the data to DNA repositories (20), implementing preventive and predictive medicine.

All these tasks should be performed fulfilling the legal, technical, and financial frameworks of the national eHealth ecosystem, without diverting from the citizen-centered objective (12). To manage that, citizens should be able to give their consent to any biomedical study that they would like to participate in and to state the period of participation [e.g., broad consent forms (23)]. It is encouraging though that citizens are willing to enroll in such processes when they feel trust and that their privacy is protected as well as when they see that related issues that may arise are successfully addressed (24). An integrated national eHealth ecosystem built with these principles and bridged with an effective communication strategy about a citizen's benefits that arise from his/her participation in such studies can increase the overall population's enrolment of the studies (4).

### An Overview of Electronic Health Record Linked Biobanks

Recently, there is a plethora of biobanks, mentioned in the current bibliography, that work in partnership with national or private EHR systems. Comprehensive studies examining the established EHR-linked biobanks can be found in (19, 25, 26). A summary of the most popular biobank projects working with EHR is presented in Table 1.

**Table 1:** A selection of major biobank schemes that work with EHRs.

**Biobank**	**Country**	**Population-based biobank**	**Link to national EHR**	**Data types**	**References**
eMerge Network https://emerge-network.org/	USA	False	False	Genotype/WES/WGS	(3)
Million Veteran Program (MVP) https://www.research.va.gov/mvp/	USA	False	False	Genotype/WES/WGS	(27)
DeCODE Genetics https://www.decode.com	Iceland	True	False	WGS	(28)
BioVU https://victr.vumc.org/biovu-description/	USA	False	False	Genotype	(29)
Michigan Genomics Initiative https://precisionhealth.umich.edu/michigangenomics/	USA	False	False	Genotype	(26, 30)
BioMe^™^ https://icahn.mssm.edu/research/ipm/programs/biome-biobank	USA	False	False	Genotype	(31)
Estonian Biobank (by Estonian Genome Center) https://genomics.ut.ee/en/about-us/estonian-genome-centre	Estonia	True	True	Genotype/WES/WGS	(23, 32)
Biobank Japan http://www.ims.riken.jp/english/projects/pj02.php	Japan	True	False	Genotype	(33)
China Kadoorie http://www.ckbiobank.org/site/	China	True	False	Genotype	(34)
DiscovEHR http://www.discovehrshare.com	USA	False	False	WES	(35, 36)
UK Biobank (UKBB) http://www.ukbiobank.ac.uk	UK	True	True	Genotype/WES	(37, 38)
Genomics England https://www.genomicsengland.co.uk/	UK	True for rare disease and their families, and patients with cancer	True	WGS	(39)
Generation Scotland: The Scottish Family Health Study (GS:SFHS) https://www.ed.ac.uk/generation-scotland	Scotland	True for families cohort	True	Genotype	(40–42)

*WES, whole-exome sequencing; WGS, whole-genome sequencing*.

The UK (including England, Wales, Scotland, and Northern Ireland) and Estonia seem to be two of the few countries that offer a single-payer-and-provider comprehensive healthcare system (43) covering the total of their population. As observed from Table 1, both of them have population-based biobanks coupled with a national EHR system. However, the coupling process is a continuously evolving ongoing process that is not completed for any of them. All other biobanks presented in Table 1 are not linked with a national EHR system.

Yet all EHR-based biobanks of Table 1 have made remarkable progress in EHR linking processes, which are worth to study and adopt/extend the best practices created and used by these leading initiatives and consortia. Moreover, the different challenges that these studies have faced in genomic test interpretation, understanding, and communication must be examined. For instance, issues related to missing data and lack of quality have been reported (26). This is a common problem in EHR-based biobanks and can happen for a variety of reasons. For example, specific tests are ordered only for specific disease-diagnosed citizens, while healthy people's EHR lack this information. Statistical and machine learning mechanisms are employed to deal with this problem (44–46). Actually, the rapid acceleration of statistical, computational, and machine learning method development urges the need to define their utilization perspective in genomics-aware EHRs (19). Furthermore, the development of a robust sustainable ethical/legal/social framework of the EHR-integrated biobanks must be formalized (5). Another open topic of discussion is about the potential bias inherited in EHR health data and how this bias confounds the analysis of biomedical studies (8). Bias related to EHR can be expressed by the loss to follow-up or the absence of clinical information for a study participant (21). EHR information bias may also apply for different data collection methods that can be used to record a specific health data measurement amongst different health providers (47). A critical challenge, which applies for the whole spectrum of biobanks, especially the EHR-based biobanks, at the international level is the establishment of data harmonization processes amongst biobanks (8). The need for harmonizing clinical sequencing and interpretation has been identified by the eMERGE Network and set as target for the network's phase III. By the end of 2019, they managed to harmonize two sequencing centers toward the technical and interpretive aspects of the clinical sequencing tests (48). Another effort for the establishment of globally scalable technology, policy, and procedures regarding the sharing of biospecimen and phenotypic data on wide consented cohorts has been proposed by Mandl et al. (49). Finally, a research topic of broad and current interest is the creation of interoperability standards describing management and sharing of genomic and clinical data amongst EHR-paired biobanks (2, 32). Although the importance of introducing interoperability in EHR (12) and in biobank systems (50) is recognized by the main stakeholders of healthcare systems, the actual progress toward true semantic interoperability has been slow, even for well-developed national healthcare information systems (51). “Enabling genomic data sharing for the benefit of human health” is the motto of the Global Alliance for Genomics and Health (GA4GH) network whose strategic plan involves progress acceleration of standards and frameworks for genomic data sharing aiming in responsible sharing of clinical-grade genomic data by 2022 (52). Ultimately, the employment of current and emerging interoperability standards that will enable EHRs' understanding of genetic/genomic data and biobanks understanding of clinical and phenotypic data might be the only way to reach a complete integration between EHR systems and biobanks.

### The Impact of the Proposed Integrated eHealth Architecture Linking Electronic Health Records to Biobanks

Dealing with the challenges of using EHR data in research and linking them to biobanks will enable the systematic pairing of clinical studies (both observational and interventional) and healthcare provider-initiated examinations with the biobank specimens data for

- cost-effective longitudinal studies in genomic medicine through the utilization of the longitudinal character of the EHR data;
- theoretically lifetime duration of clinical trials and studies, which could observe the outcome of participants through EHRs for as long as the participants provide consent, without the need or cost of follow-up visits; and
- improvement of personalized clinical care by considering genetic/genomic implications to patient care throughout their clinical workflow to reach a clinical decision.

The novelty of this proposal is that both the proposed national integrated EHR and the population-based integrated biobank are based on two research projects, eHealth4U and CY-Biobank, which have recently initiated and they will have a nationwide impact in Cyprus. The eHealth4U project has initiated the design and the development of a national integrated EHR system where the CY-Biobank is designed to be a major national health resource aiming in supporting applied and basic research for improving health quality. The fact that the two projects run in parallel gives them the potential to incorporate, from the very beginning, the required EU and national standards and protocols in their structural design and to find the most suitable integration architecture in the context of the eHealth national ecosystem. To do that, technological, legal, and interoperability elements must be crystallized in deep detail. A very first approach is presented in the following sections.

## Proposed Integration Framework eHealthBioR

The objectives of eHealthBioR based on the policy modules of the framework are as follows:

- to support the efficient and near-real-time calculation of statistical power for prospective experiments, including providing incidence and prevalence of conditions utilizing privacy-preserving aggregate data approaches;
- to support monitoring linked to policy enforcement through centralized dashboards to ensure data security, ethical use of data, and support efficient policy evolution;
- to enable the automated long-term monitoring of consenting citizens' EHRs to research projects they have approved and tonsure national, international (GDPR) privacy legislation;
- to ensure secure and transparent data communication, storage, and analyses;
- to generate reports available to participating citizens on the use of their data across multiple studies, while enabling them to exercise their rights at any point in a way that builds confidence and supports engagement of citizens/patients in research; and
- to support in the long term the creation of participatory design studies that start from the main stakeholders, the patients, and to ensure that researchers focus on objectives prioritized by the patients and that the research maintains its focus on reaching conclusions maximizing the positive impact to patients.

**Figure 1:**
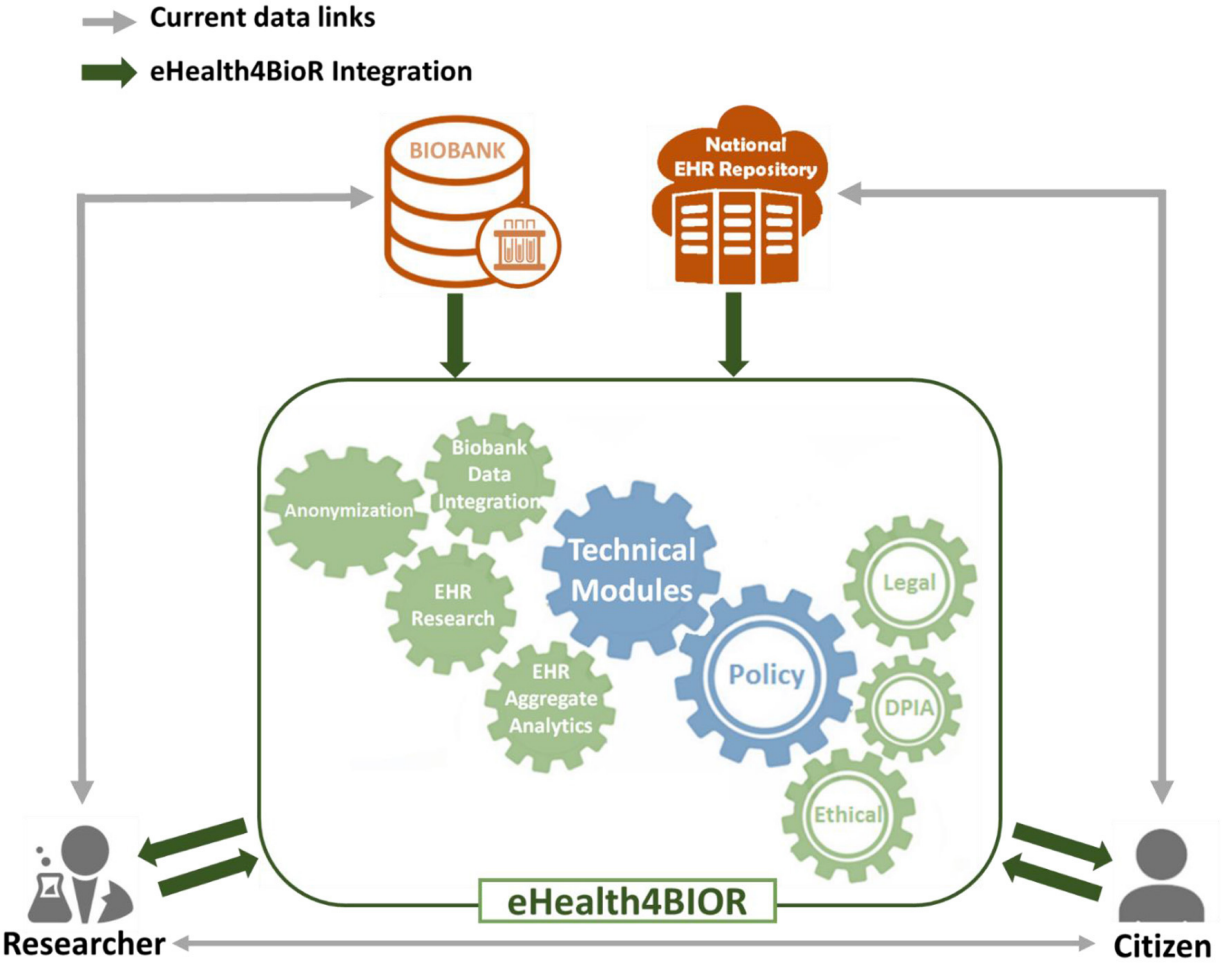
eHealthBioR framework.

Figure 1 presents how researchers in the biomedical domain are currently working regarding the design and implementation of their research studies in contrast to the way that they are expected to handle their research studies in the eHealthBioR framework. More precisely, nowadays in Cyprus, an authorized researcher has access only to the existing biobanking data of a citizen who participates to a research study (gray line linking the researcher and biobank in Figure 1). With the implementation of eHealth4U (expected to finish in 2022), all the clinical healthcare data of the citizen shall be kept in the national EHR repository of Cyprus and will be under the control of the citizen (gray line linking the citizen and national EHR repository). However, with the current schema, if clinical healthcare data are required for the study, the researcher must contact the citizen in person to extract the extra clinical information needed (gray line linking the researcher and citizen). A different approach is proposed by the eHealthBioR framework, which will allow the registration and implementation of research studies (green arrow between the researcher and the eHealthBioR rectangle). The citizen can join the system and select to participate to a research study by giving an informed consent and then can monitor how the research study progresses through time (green arrows between the citizen and the eHealthBioR rectangle). The informed consent of a citizen dictates which citizen's data can be pulled from the two connected repositories, the population-based biobank and the national EHR Registry presented with green arrow between the two repositories and the eHealthBioR rectangle in Figure 1. Pulled citizen's data will then be provided to the selected research study for analysis and further processing using the technical modules while addressing legal and ethical challenges, enabling research, and empowering citizens to participate.

In the current model, researchers can apply to gain access to data through a biobank, and biobanks enroll patients through their healthcare providers or directly. Researchers may also collaborate with healthcare providers and also gain access to data or subjects for prospective studies through that interaction. However, in both cases, privacy-related legal and ethical challenges must be met, and this poses a significant threshold, that to overcome, and enable efficient sharing; in many cases, anonymization methodologies are chosen to limit or eliminate the possibility for the researcher to gain access to the patient's personal information. This introduces hurdles in the process, such as the need for the healthcare provider to de-anonymize data and evaluate legal and ethical aspects including risks increasing the cost. But most importantly, this approach works for collecting prospective data defined as part of clinical research forms, samples collected, etc., but it does not enable easy direct access to prospective data, anyway collected by healthcare professionals in EHRs. Furthermore, there is no universal way of enrolling citizens across multiple healthcare providers or gaining access to all data referring to subjects that are provided with healthcare by different carers (such as the case of comorbidities and the need to access different providers for specific conditions).

eHealthBioR sits in the middle of researchers, citizens, biobanks, and the National Electronic Health Registries, providing the relevant modules to both integrate the data sources, clean, and quality control, as well as the tools needed to ensure legal and ethical adherence and data access to both researchers and citizens.

### The Case of Cyprus Biobank and Electronic Health Record Integration

Cyprus, being a small country (population 1 million) that was at the same time initiating a planned large biobank, as well as designing a national integrated EHR system, provided a unique opportunity to achieve the implementation of the proposed integration framework to act as a case study. The implementation of the prototype of the national integrated EHR system was initiated in October of 2019 (eHealth4U). At the same time, the design and the implementation of a population-based biobank (Cy-Biobank) in Cyprus also began. These two systems are expected to have a strong impact on Cypriot citizens' healthcare services. Additionally, researchers can also benefit from the deployment of these two systems, since they will be able to access biobanking and clinical information for the same citizen avoiding to ask the citizen to repeat questions, examinations, measurements, or any other healthcare information that is already included in the citizen's national integrated EHR or biobank profile. The proposed framework is the result of this work, and although it is designed as a generic framework able to be re-purposed across different national and international platforms, it is currently undergoing development based on the specific requirements of Cyprus. The ultimate objective is the production of reports demonstrating the specific cost, benefits, and challenges faced to support the development of platforms in other countries and settings.

The fact that the proposed framework involves the national integrated EHR, which is not just another commercial EHR system, should be considered as a very important factor for the success of the proposed framework. The national integrated EHR system will contain the minimum set of healthcare data required by the national medical associations in Cyprus to describe the health status of a citizen. The healthcare data will be given in an interoperable format, allowing other healthcare systems to communicate and exchange the total of the citizen's healthcare information. The contents of this core set of health data shall be determined by a decree of the Minister of Health, and all healthcare providers serving in Cyprus will be obligated to update in a responsible manner the EHR profiles of their patients.

The holders and operators of the healthcare systems and databanks will be regulated by the NeHA, which was established by law in 2019.^1^,^1^ The national eHealth law defines the legal framework under which the healthcare providers will work. As stated in the relevant law, the NeHA will be responsible to monitor their compliance with the relevant national and European Union laws and relevant standards but also with the national eHealth objectives.

Consequently, the proposal of this paper is about the development of the underlying framework (eHealthBioR), which takes for granted the existence of the following:

- a national integrated EHR system with a Single e-Health Records Bank^1^;
- a population-based biobank with a Healthcare Provider's Databank^1^; and
- a regulator monitoring all processes regarding healthcare data and databanks^1^.

The fashion in which data are collected, stored, deleted, or retrieved for the Single e-Health Records Bank or Healthcare Provider's Databank is out of the scope of this paper.

The target of the eHealthBioR is to facilitate the implementation of research studies by providing access to healthcare data describing the same citizen pulled from both systems, the national integrated EHR Single e-Health Records Bank and the population-based biobank databank. Besides, as reported in (22), the requirement of providing (by the citizen) a specific consent for every research study that uses biological materials or personal data obtained during a medical intervention is stated in the Protocol on Biomedical research states^2^ and confirmed in the EU Clinical Trials Regulation (No. 536/2014 EU)^3^.

Based on this requirement, the eHealthBioR provides the following services to the citizen:

- View ongoing and future research studies that ask for participants.
- Become informed about each suggested research study at any time from any place for as long as he/she wishes. Per each research study, the following information will be available (53):
  - purpose(s) of the research study,
  - a description of any reasonably foreseeable risks or discomforts to the subject or the society,
  - a description of any benefits to the subject or to others that may reasonably be expected from the research,
  - a statement describing the extent, if any, to which confidentiality of records identifying the subject will be maintained,
  - whether the results of the research study might be used for commercial purposes,
  - entities involved in the research study (e.g., universities, laboratories, and pharmaceutical companies),
  - relevant legislations, EU directives, or any other legal or ethical binding regulations that characterize the research study,
  - research study phases indicating the steps taken in the context of each phase and the time points that a citizen can join the study, and
  - report explicitly the healthcare data that the research study asks for authorization to gain access (from both sources—EHR and biobank).

The eHealthBioR framework will allow the employment of multiple electronic media, including text, graphics, audio, video, podcasts, passive and interactive Web sites, biological recognition devices, and card readers, to convey information related to the study.

(c) Provide informed consent to a research study. The informed consent must be created following the guidelines given by European Commission in Ethics Review in FP7: Guidance for Applicants.^4^ The effective and expiry dates of the consent will be clearly indicated. The citizen will be also informed about the right to revoke the consent at any time.
(d) View the studies that he/she participates in and the granted access rights.
(e) Monitor the progress of the research studies that he/she participates in. The citizen will have access to the research studies results, publications, media and press, commercialization of the results, etc.
(f) Revoke the consent from a research study that he/she participates in.

The citizen can do all the above from the comfort of his/her home or any other desired place, at any time, without feeling pressured by any person, place, and time limitations. The citizen becomes the key component of the research leading to the production of citizen-centered research with self-administered participants being informed about the research before their registration and continue learning about how the research develops through the whole lifetime of the study (given that the citizen does not withdraw the consent). The proposed framework perceives the citizen as the only controller of his/her own healthcare data (either stored in a biobank or the national integrated EHR) and the decision whether any other entity (e.g., researcher, research organization, and hospital) will be provided with access rights to a subset of his/her data belongs exclusively to the citizen. This is fundamental for the eHealthBioR framework in order to empower the citizens and thus create the potential to become a leading tool designed to optimize health research and citizen-centered care in practice. In that way, it is expected to increase citizens' enrollment rates to research studies as well as their recruitment and retention improving at the same time the communication of the research progress and development to the wider public educating citizens toward new scientific information on health and biomedical topics (e.g., treatments, cures, and prognosis). Therefore, while the main objective of the eHealthBioR is to create a wide range of capabilities for better understanding, increased motivation, and high engagement for the citizen, the researcher also benefits from it by increasing the efficiency of research implementation and improving the research quality.

The researcher entity (human or organization) is capable to do the following when using the eHealthBioR:

- Register a new research study in the eHealthBioR framework by providing the context of the research study specific informed consent that will be provided by the citizens that wish to participate:
  - provide all the information required as presented in the eHealthBioR services to the citizen, point (b, i–ix);
  - specify if the study requires identifiable subjects and to what extent;
  - set minimum number of participants;
  - set maximum number of participants; and
  - set the duration of the study.
- For each of the registered research studies:
  - view the list with the participants who provided the study-specific informed consent. If the study-specific informed consent defines anonymized subjects, then the list will be given in a format that will retain the anonymization feature; and
  - access (read only) the healthcare data as explicitly declared in the study-specific informed consent provided by the citizen-participant.
- Close a research study, and, therefore, all the study-specific informed consent given by the participants will be revoked.

Acknowledging the complexity of designing and implementing a research study that combines biobanking data with clinical healthcare data, the challenge of the eHealthBioR is to provide a trustful environment for the citizen in which he/she will feel safe and confident to provide the researcher entity with access rights to his/her data by an intentional choice. The key factor to success is to provide the right tools to build the research study specific informed consent and the proper function of the framework ensuring that all processes using citizen's data are compliant with the context of the provided informed consent.

**Figure 2:**
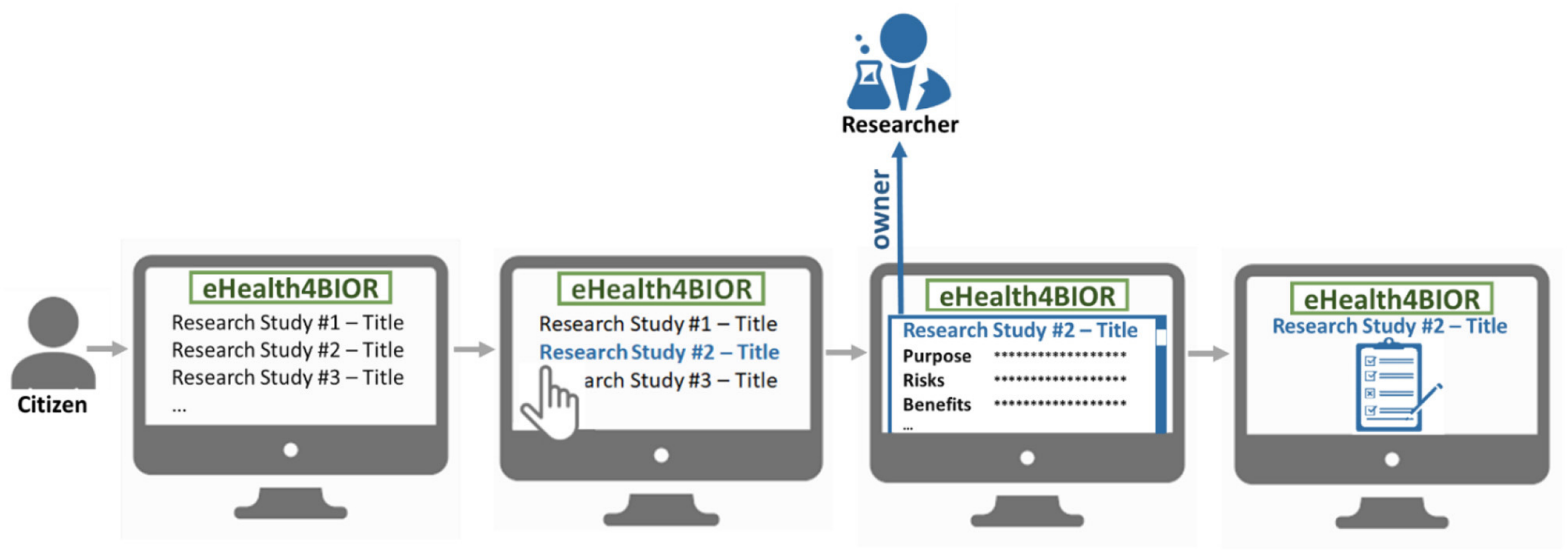
The process of the voluntary participation of a citizen to a research study under the eHealthBioR framework.

The citizen can join a research study following an opt-in model, as illustrated in Figure 2. In the eHealthBioR framework, each citizen has access to view information about all the active research studies, which are registered in the system by researchers at that particular time period. The citizen shall be able to select a research study he/she is interested in and study the information that is important for the citizen to know in order to provide an informed consent. Then, voluntarily, the citizen can provide the informed consent for the selected research study, which will enable his/her participation to the study.

**Figure 3:**
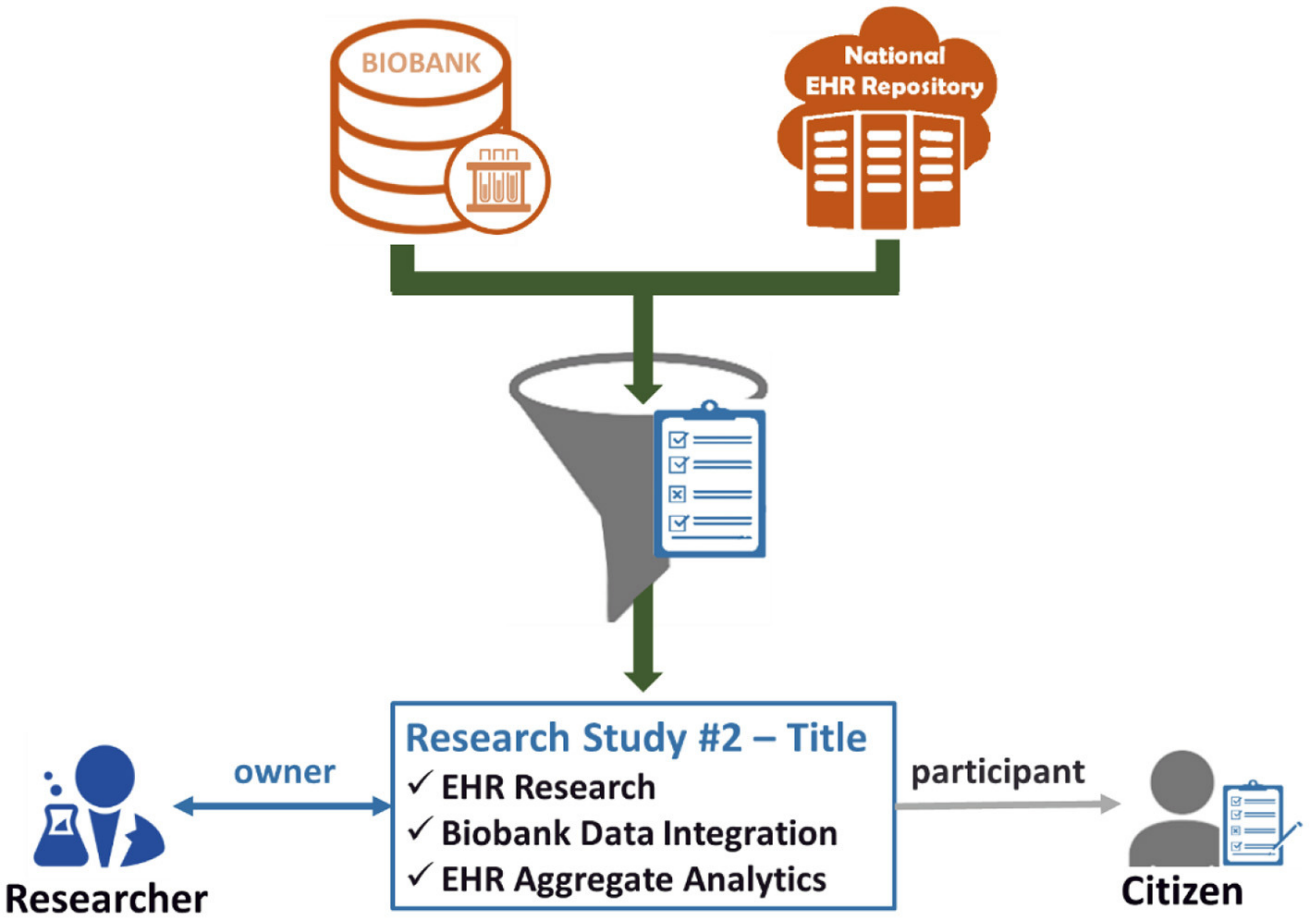
Citizen's biobanking and national electronic health record (EHR) data flow controlled by the citizen's informed consent.

The last step leads to the creation of an active consent, which will define explicitly what citizen's data can be drawn from the biobank and the national integrated EHR Repository to be used for the referenced research study for which the researcher is the owner and the citizen is the participant. Figure 3 exhibits the importance of the active consent provided by the citizen to a researcher for the purposes of a specific research study. Whenever the researcher wishes to process the citizen's data with the technical modules provided by the eHealthBioR framework (Figure 1), the active consent will be used to form the final set of the biobanking and clinical citizen's data retrieved from the two repositories as presented in Figure 3. In the same figure, the researcher appears to interact with eHealthBioR since he/she will be able not only to view but also to process the citizen's dataset by using the technical modules provided by the eHealthBioR framework. Accordingly, the citizen is presented to receive information from the eHealthBioR, which allows a participant of a particular research study to follow and get updates on the results, the outcomes, and any other related activity.

The implementation of the eHealthBioR e-consent must be designed in such a way as to promote the participating researchers to fulfill the general requirements of a study-specific informed consent (53) using understandable language to provide the relevant information. At the same time, they should be promoted to avoid the use of exculpatory statements, which waive or appear to waive the participant's legal rights.

### The Electronic Health Record Research Module

EHR-sourced data represent a national pool of citizens' phenotyping information that represents the state of the health of citizens through time. Biobanks on the other hand act as biospecimens warehouse coupled with research quality data, which can be used to implement biomedical studies toward preventive, predictive, and therapeutic knowledge discovery, or intervention/diagnostic testing. Still, clinical information is very important for biobanking studies, and therefore, biobanking data should be linked to citizen's EHR data. In that way, biobanks gain access to a huge amount of up-to-date information about the participants to their studies, creating holistic datasets to address complex diseases and conditions and enabling long-term monitoring in longitudinal studies with minimal cost. The EHR and biobank integration model offers a tremendous opportunity for the biobanks to explore health information for the citizens of their cohorts for their life beginning 9 months before their birth until their current age or their death.

The EHR research module will enable the direct access of relevant data of subjects who have provided an informed consent for an approved study longitudinally, designed as a method to monitor patients in the long term. Nevertheless, the patients should be informed about how and what their data are used for and enable them to withdraw their consent at any point through the national EHR platform. This module can work as a catalyst supporting research activities such as biobanking, by both enabling the longitudinal monitoring of the patient through their EHRs and by enabling the user to have knowledge of when, how, and why their data are used with the ability to withdraw consent at any point.

This will include an API that will allow the long-term monitoring of the patients through the EHRs for research purposes while empowering the patient with the ability to withdraw consent or exercise any of their other rights as a subject in the study at any time through the system.

The API will authenticate the research entity requesting the data and only provide it access to citizens who have completed all policy requirements to participate in that study and only data that have been approved as part of the relevant informed consent and bioethics approval.

### The Electronic Health Record Aggregate Analytics Module

The aggregate analytics module will perform in near-real-time aggregation and preliminary statistical analyses of all data in the EHR system focusing on disease prevalence and incidence. As this module will only provide aggregate data, the generated outcomes will not constitute personal data, and therefore, the requirements for access to data generated by this module will be limited. Care will be taken to ensure de-anonymization is not possible by utilizing well-established methods including perturbation and data suppression (16, 18).

This will support governmental organizations at the national (Ministry of Health) as well as the international (World Health Organization) to have direct access enhancing their activities, and early identification of issues, such as changes in the incidence of infectious diseases. Furthermore, it will help guide research by the identification of the most prominent research challenges (diseases with sudden recent increases or high overall incidence). Additionally, it is easier to identify the subjects that could be informed of a future research and asked to provide their consent to participate, provided that the legal and ethical challenges are addressed through proper channels.

### The Biobank Data Integration Module

Currently, there are independent legacy EHR systems across different healthcare providers; however, the number of these that support international standards for encoding and communication is increasing with support mandated at the policy level across many countries.

To achieve integration with EHRs on a biobank's side, bi-directional communication protocols need to be developed through secure APIs. This module will support both the pulling of data from existing external to the biobank EHR systems and the integration of these data with the biobank's own sample databases. This process will enable the long-term monitoring of the patient and will provide bi-directional communication informing the EHR system (if it supports this functionality) with what data have been produced from the biological sample provided to the biobank, focusing on data that are or may become in the near future relevant to the healthcare provider's team. Examples include somatic or cell line mutations in cancer and patients detected through sequencing of samples provided to the biobank that, although performed as part of research projects, may help identify optimal secondary treatments. This will enable the biobank to enrich the data of patients who provide samples.

## Discussion

There is great research potential in EHR records, but there are also key challenges. These challenges can all be traced back to the purpose of EHRs, that is, to support the individual citizens' healthcare and to some extent health insurance provision not to enable research. Already multiple research initiatives have successfully demonstrated that EHRs can and should be used in research, as they provide a low-cost unique longitudinal data source spanning potentially the entire life of the subjects in the studies. It is also been demonstrated that it is possible to address to a sufficient extent the challenges that EHRs pose to be reused in research, related to missing data, quality of data, or the diverse potential sources of data. However, to ensure legal and ethical compliance, without any data loss, and to simultaneously enable the integration of data from different sources of the same subjects (such as the case of patients providing samples and data to biobanks), following techniques of anonymization is the wrong approach. By definition these rely on destroying the link between the data and the individual, but that is what we most want to preserve in order to link the EHR to the structured electronic case report forms (eCRFs) and data collected through the biobank.

Thus, what is proposed in this paper is an approach that puts the citizen-patient in the driver's seat, enabling them to quickly review and take informed decisions about providing consent for specific research experiments, monitor the use, and empower them to practice their legal rights. This way, research participants are empowered to join, maintain their interest in the study, and understand how, when, and why their data are used. Most importantly, they can engage with the research in a meaningful way, including observing potential research outcomes relevant to their care in the long term. We suggest that this eHealthBioR or similar integrations of biobanks to EHR registries may act as a key enablers in engaging more citizens, healthy or patients, in research, while also increasing adherence to the study directions.

## Conclusion

In this paper, we outlined the state of the art in data-reuse from EHRs and analyzed that although challenging to use in research, EHRs pose a tremendous opportunity, especially for long-term longitudinal studies. However, current approaches that attempt to solve the privacy aspects through anonymity or federated executions, suffer from a major disadvantage in that they do not allow for external to the EHR registry data silos that contain information on the same subjects to be integrated in the EHRs. Furthermore, the legal and ethical challenges associated with access health data, especially in the case of molecular level data such as genetics that are inherently personal data, pose a significant hurdle to research. We proposed a methodology that addresses these challenges, and we suggest a framework that integrates a national integrated EHR system with biobanks within a legal and ethical context to support the citizens to provide their data for research purposes. In this way, the strict adherence to legal and ethical requirements is ensured as well as the empowerment sense of the participants since they are enabled to exercise their legal rights. We provide a first draft of how the integration of such modules could be achieved through the proposed eHealthBioR.

## Data Availability Statement

The original contributions presented in the study are included in the article/supplementary material, further inquiries can be directed to the corresponding author.

## Author Contributions

All authors listed have made a substantial, direct and intellectual contribution to the work, and approved it for publication.

## Conflict of Interest

The authors declare that the research was conducted in the absence of any commercial or financial relationships that could be construed as a potential conflict of interest.

## Footnotes

^1^No. 59 (I)/2019 Electronic Health Law of Cyprus.

^2^Explanatory Report, CETS 195, 78.

^3^Art. 28.2, EU Clinical Trials Regulation EU No. 536/2014.

^4^EUROPEAN COMMISSION, Ethics Review in FP7: Guidance for Applicants: Informed Consent. ftp://ftp.cordis.europa.eu/pub/fp7/docs/informed-consent_en.pdf.
